# Comparison of Physicochemical, Microbiological, and Sensory Properties of Yogurt Made From Cow, Goat, Sheep, and Buffalo Milk with and Without Probiotic Cultures

**DOI:** 10.1002/fsn3.71743

**Published:** 2026-04-15

**Authors:** Şevval Şevgin Demirhan, Seval Andiç, Şehriban Oğuz

**Affiliations:** ^1^ Institute of Natural and Applied Sciences Van Yuzuncu Yil University Van Turkey; ^2^ Faculty of Engineering, Department of Food Engineering Van Yuzuncu Yil University Van Turkey

**Keywords:** Bifidobacteria, milk type, physicochemical properties, probiotics, sensory evaluation, yogurt

## Abstract

This study investigated the physicochemical, microbiological, textural, and sensory characteristics of yogurts produced from cow, goat, sheep, and buffalo milk, both with and without probiotic supplementation ( *Lactobacillus acidophilus* LA‐5 and *Bifidobacterium animalis* subsp. *lactis* BB‐12), during 21 days of refrigerated storage. Results revealed that milk type markedly influenced compositional and structural attributes (*p* < 0.05). Sheep and buffalo milk yogurts exhibited higher dry matter, viscosity, and hardness, while goat milk yogurts showed the weakest water‐holding capacity (WHC) and highest syneresis. The addition of probiotic cultures modified fermentation dynamics, leading to lower acidity and higher pH values, particularly in bifidobacteria‐enriched samples. All probiotic yogurts maintained viable counts above 10^6^ CFU/g throughout storage, confirming their probiotic status. Interestingly, goat milk provided the most favorable environment for *B. animalis* subsp. *lactis* BB‐12 survival, likely due to its higher oligosaccharide content and buffering capacity. Sensory scores were highest for sheep and buffalo milk yogurts, reflecting their richer composition and superior texture. These findings highlight the strong influence of milk composition on the physicochemical behavior and probiotic viability of yogurt, suggesting that mixed‐milk formulations could improve the sensory and functional quality of probiotic dairy products.

## Graphical abstract

This study demonstrated that milk type, probiotic culture, and storage period significantly influenced the physicochemical, textural, microbiological, and sensory properties of yogurt. Sheep and buffalo milk yielded yogurts with superior structural and sensory quality, whereas goat milk provided the most favorable environment for probiotic survival, particularly *Bifidobacterium animalis* subsp. *lactis* BB‐12. Correlation analysis revealed strong relationships between texture, water‐holding capacity, acidity, and starter and probiotic viability, providing mechanistic insight into yogurt quality development.

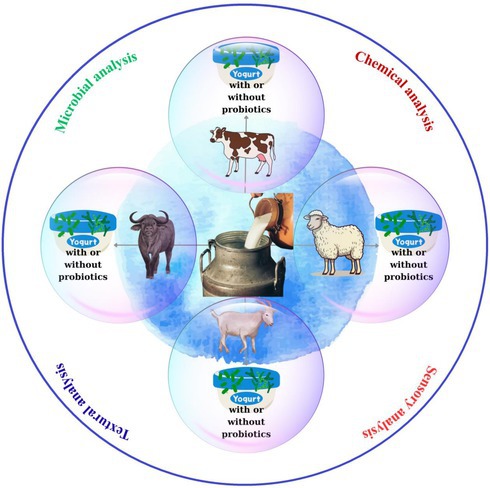

## 1 Introduction

Milk is a nutrient‐rich secretion of the mammary glands in mammals, primarily produced to nourish offspring during the early stages of life until the transition to solid foods. It contains a balanced composition of nitrogenous compounds, lipids, carbohydrates, vitamins, and minerals that meet essential nutritional requirements (Yüksel‐Önür 2015; Pietrzak‐Fiećko and Kamelska‐Sadowska 2020; Arrichiello et al. 2022). Although the fundamental constituents of milk are similar across mammalian species, their relative proportions vary depending on species, feeding regime, environmental conditions, and physiological status of the animal (Arrichiello et al. 2022; Gross 2022). These compositional variations directly influence the physicochemical characteristics of milk and, consequently, its technological functionality and suitability for the manufacture of fermented dairy products.

Differences between the milk of ruminant and non‐ruminant animals are particularly pronounced. Milk from ruminants such as cow, sheep, and buffalo is characterized by higher protein and fat contents, whereas non‐ruminant milk, including that of horse and donkey, contains higher levels of lactose and oligosaccharides and shows greater similarity to human milk in this respect (Agnihotri and Prasad 1993; Licitra et al. 2019; Pietrzak‐Fiećko and Kamelska‐Sadowska 2020; Martini et al. 2021; Arrichiello et al. 2022). Because of these compositional characteristics, together with their high production volumes, ruminant milks constitute the primary source of milk consumed worldwide (Roy et al. 2020a).

Among ruminants, buffalo, cow, sheep, and goat milk are widely utilized in the production of fermented dairy products such as yogurt, kefir, and cheese. These milk types differ considerably in terms of dry matter, fat, and protein contents. Buffalo, sheep, and goat milks generally exhibit higher dry matter and protein levels than cow milk. Furthermore, while buffalo and sheep milk proteins contain higher proportions of casein, goat milk proteins are characterized by relatively lower casein and higher serum protein contents (Roy et al. 2020a; Arrichiello et al. 2022). Such compositional differences are critical determinants of fermentation performance, texture formation, and sensory quality in yogurt production.

Lactose serves as a major energy source, particularly for brain development, and also constitutes a structural component of complex milk oligosaccharides (Engfer et al. 2000; Coppa et al. 2006). The lactose contents of cow, goat, sheep, and buffalo milk range between 44–56, 41–59, 32–50, and 32–49 g/L, respectively (Park et al. 2007; van der Toorn et al. 2023). Milk oligosaccharides play crucial biological roles, including modulation of intestinal microbiota, stimulation of immune responses, and protection against pathogenic infections (Engfer et al. 2000; Coppa et al. 2006). While human milk contains oligosaccharides at concentrations 100–1000 times higher than those found in ruminant milk, notable differences exist among animal species. Goat and buffalo milk exhibit higher oligosaccharide contents than cow and sheep milk, and the bioactivity of goat milk oligosaccharides has been reported to be comparable to that of human milk oligosaccharides (Agnihotri and Prasad 1993; Barłowska et al. 2011; Bode 2012; Licitra et al. 2019; van Leeuwen et al. 2020; Garau et al. 2021).

In recent years, increasing attention has been directed toward enhancing the functional properties of fermented dairy products through the incorporation of probiotic microorganisms. Species belonging to the genera *Lactobacillus* and *Bifidobacterium* are among the most dominant beneficial microorganisms in the human gastrointestinal tract and are widely used in commercial probiotic products (Chen et al. 2017). In particular, *Lactobacillus acidophilus* LA‐5 and *Bifidobacterium animalis* subsp. *lactis* BB‐12 have been extensively studied and shown to survive gastrointestinal conditions and exert beneficial effects, including modulation of intestinal microbiota, immune regulation, and antimicrobial activity (Isolauri et al. 2000; Merenstein et al. 2015; Nowak et al. 2018; Ba et al. 2021; Melsaether et al. 2023).

The physicochemical composition of milk is a key factor influencing fermentation kinetics, texture development, microbial viability, and sensory attributes of yogurt. Therefore, the selection of milk type, together with the choice of probiotic culture, plays a critical role in determining the quality and functionality of the final product.

Accordingly, the present study aimed to investigate the effects of milk type (buffalo, cow, sheep, and goat), probiotic culture ( *L. acidophilus* LA‐5 and *B. animalis* subsp. *lactis* BB‐12), and storage period on the physicochemical, microbiological, textural, and sensory characteristics of yogurt. The viability of probiotic strains and changes in quality parameters were monitored during refrigerated storage at 4°C for 21 days.

## 2 Material and Methods

### 2.1 Material

**FIGURE 1:**
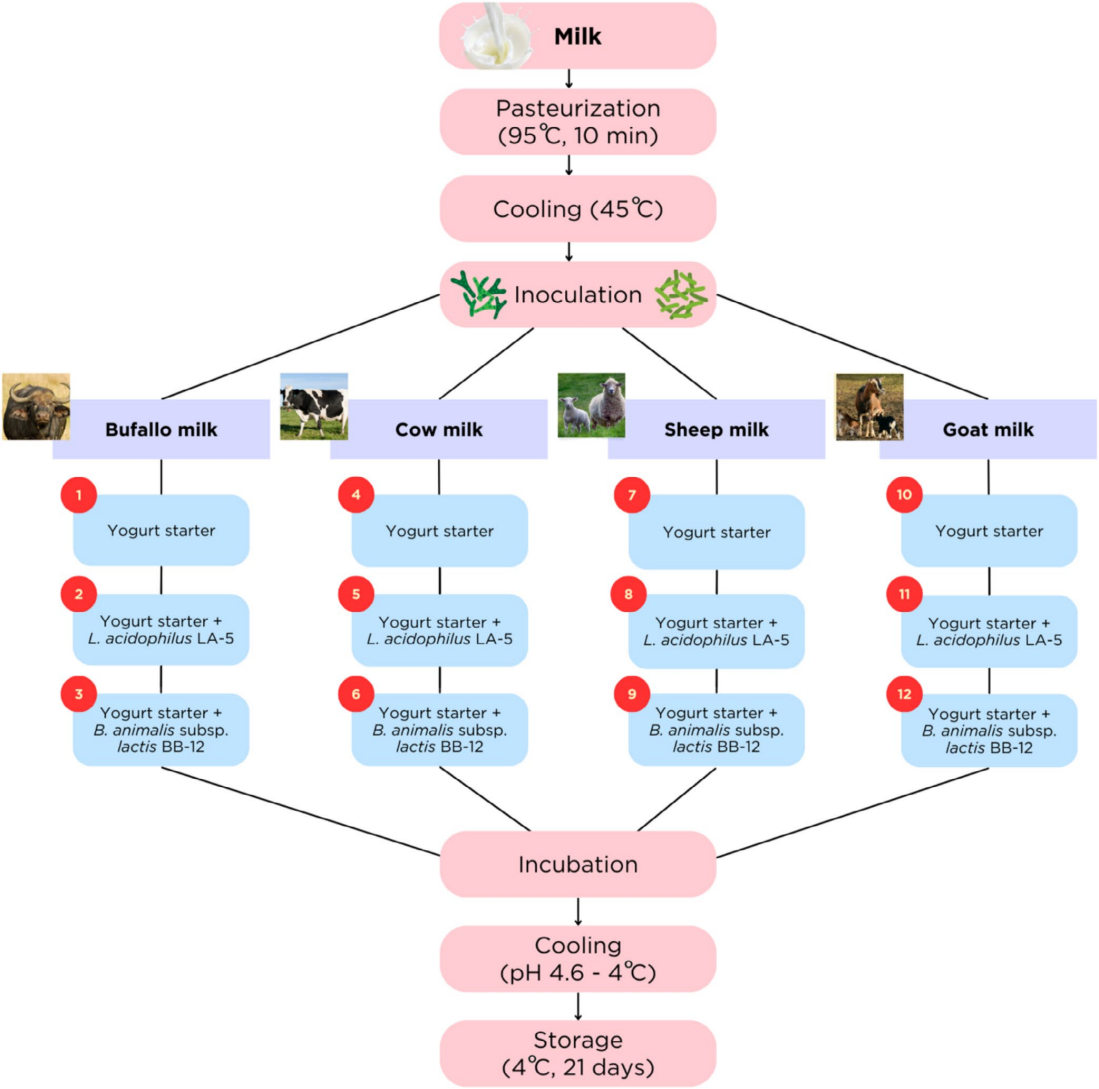
Production steps of yogurt samples.

Buffalo, cow, sheep, and goat milks were obtained from local family farms in Van Province, Türkiye. A commercial yogurt starter culture was obtained from Maysa and probiotic strains ( *L. acidophilus* LA‐5 and *B. animalis* subsp. *lactis* BB‐12) were sourced from Chr. Hansen (Denmark). Each type of milk was pasteurized at 95°C for 10 min, cooled, and then divided into three portions: (1) inoculated with yogurt culture, (2) inoculated with yogurt culture + *L*. *acidophilus* LA‐5, and (3) inoculated with yogurt culture + *B. animalis* subsp. *lactis* BB‐12, at an initial probiotic concentration of approximately 10^8^ CFU/g. Samples were incubated at approximately 43°C until the pH reached 4.6, then stored at 4°C for 21 days (Figure 1).

### 2.2 Physicochemical Analyses

Dry matter, fat, and acidity values were determined using the methods described by Hooi et al. (2012). A calibrated pH meter (Thermo Scientific, USA) was used to measure pH. Water‐holding capacity was measured by centrifuging 2 g of yogurt at 13,500 × *g* at 10°C for 30 min (Parnell‐Clunies et al. 1988). For serum separation, 40 g of yogurt was centrifuged at 222 × *g* at 10°C for 10 min (Keogh and O'Kennedy 1998). Values were calculated as a percentage of the initial sample weight.

### 2.3 Determination of Viscosity

Viscosity was measured at 4°C using a Brookfield DV‐III Ultra rheometer with a helipath system. Prior to measurement, samples were homogenized by stirring 10 times in each direction with a spatula. A T‐bar probe was used at 20 rpm, and data were collected every 10 s for 10 intervals.

### 2.4 Texture Profile Analysis (TPA)

Textural properties were analyzed using a TA.XT2 Texture Analyzer (Stable Microsystems, UK) equipped with a 5 kg load cell. A 25.4 mm cylindrical probe was used to perform two penetrations at a speed of 0.5 mm/s to a depth of 4 mm. From the resulting graph, hardness, stickiness, gumminess, and elasticity values were calculated.

### 2.5 Cultural Enumeration of *Streptococcus thermophilus*

M17 (Terzaghi‐M17/Merck, Germany) medium was used for the enumeration of *S. thermophilus* . The prepared medium was sterilized in an autoclave at 121°C for 15 min, then cooled to 45°C–50°C and poured into gamma‐irradiated Petri dishes as 12–15 mL. Serial dilutions of yogurt samples were prepared using 0.1% sterile peptone water. 0.1 mL of the determined dilutions were transferred onto the solidified medium and spread with a Drigalski spatula. The inoculated Petri dishes were incubated at 37°C for 72 h. At the end of incubation, suitable Petri dishes (containing 30–300 colonies) were selected for counting and logarithmic transformation was applied to the bacterial counts.

### 2.6 Cultural Enumeration of Probiotic Bacteria

MRS‐Sorbitol agar was used for *L. acidophilus* LA‐5 enumeration and MRS‐NNLP agar was used for *B. animalis* subsp. *lactis* BB‐12 enumeration. MRS medium was prepared according to the formulation and sterilized in an autoclave at 121°C for 15 min. Selectivity agents prepared in the amounts specified in the method and sterilized with a 0.22 μm MILLIPORE MILLEX GP syringe filter (Millipore, Cork, Ireland) were added to the sterilized medium, which was cooled to 45°C–50°C (Dave and Shah 1996, 1997).

For MRS‐sorbitol agar, 10 mL of selectivity agent (D‐sorbitol) was added to 90 mL of base medium, and for MRS‐NNLP agar, 20 mL of selectivity agent [nalidixic acid (50 mg/L), neomycin sulfate (100 mg/L), lithium chloride (3000 mg/L), and paromomycin sulfate (200 mg/L) (Sigma‐Aldrich, USA)] was added to 80 mL of MRS agar. L‐cysteine (0.05%) was also added to MRS‐NNLP agar to promote the growth of bifidobacteria and reduce the oxidation–reduction potential of the medium (Tharmaraj and Shah 2003).

The prepared media were poured into gamma‐irradiated Petri dishes as 12–15 mL. Serial dilutions of yogurt samples (0.1% sterile peptone water) were prepared and 2 parallel dilutions were transferred to Petri dishes. Petri dishes were incubated for 72 h in a CO_2_ incubator (10% CO_2_) at 42°C for *L. acidophilus* LA‐5 and *B. animalis* subsp. *lactis* BB‐12 (in an anaerobic jar added Gas Pak). At the end of incubation, for the enumerations Petri dishes containing 30–300 colonies were selected and logarithmic transformation was applied to the counts.

### 2.7 Viability Proportion Index

The viability proportion indexes of *S. thermophilus* , *L. acidophilus* LA‐5, and *B. animalis* subsp. *lactis* BB‐12 were calculated according to the method given by Ahmadi et al. (2012). The viability proportion index was determined as the ratio of the probiotic bacterial count at the end of the storage period to the initial probiotic count.

$$\text{Viability Proportion Index}\;\left(\mathrm{VPI}\right)=\frac{\text{Final cell population}\;\mathrm{Log}10\;\mathrm{CFU}/g}{\text{Initial celll population}\;\mathrm{Log}10\;\mathrm{CFU}/g}$$

### 2.8 Sensory Analysis

A trained panel group (*n* = 10) evaluated yogurt samples for appearance, color, odor, consistency, taste, and overall acceptability using a 9‐point hedonic scale (1 = dislike extremely, 9 = like extremely) (Aston et al. 1985). Sensory evaluations were performed on days 1, 7, 14, and 21 of refrigerated storage. For each yogurt group and sampling day, analyses were performed in triplicate, and each sample was evaluated independently by all panelists.

### 2.9 Statistical Analysis

SPSS (Statistical Package for Social Science) PASW 21 Statistical Package Program was used to evaluate the data obtained in the study. Data were analyzed using ANOVA and Duncan multiple comparison test (*p* < 0.05) to assess the effect of milk type, culture type, and storage time. All physicochemical, microbiological, and textural analyses were performed in triplicate, and the results were expressed as mean ± standard deviation.

Pearson correlation analysis was performed to evaluate the relationships between physicochemical parameters (pH, TA, syneresis, WHC), textural properties (viscosity, hardness, cohesiveness, gumminess, springiness), and microorganism counts ( *S. thermophilus* , *L. acidophilus* LA‐5, and *B. animalis* BB‐12). Correlation coefficients (*r* values) and significance levels (*p* < 0.05, *p* < 0.01) were calculated using two‐tailed tests. To visualize multivariate associations among variables, correlation heatmaps were generated based on the Pearson correlation matrix. Heatmaps were computed using the standardized correlation matrix and displayed using a color gradient representing the strength and direction of correlations (from −1 to +1). Statistically significant correlations were marked in the matrix with * or ** indicators. These analyses were used to identify clustering patterns, co‐dependencies, and mechanistic associations among milk type, structural parameters, and probiotic survival.

## 3 Results and Discussion

### 3.1 Composition of Yogurts

**FIGURE 2:**
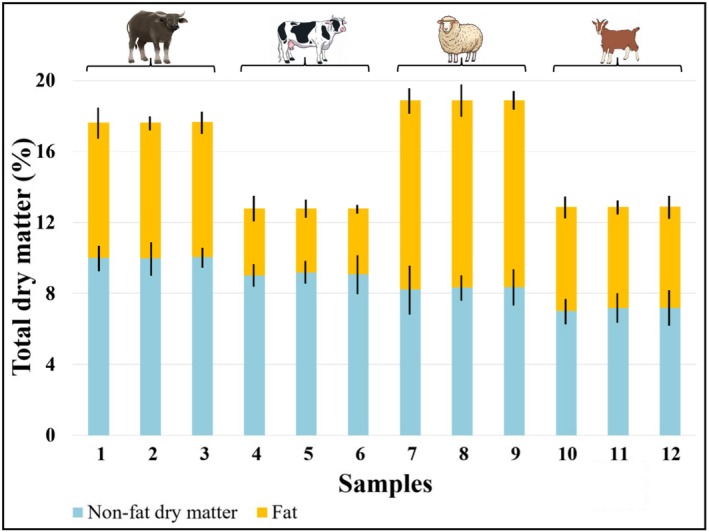
Total and non‐fat dry matter and fat values of yogurt samples. 1: Buffalo yogurt without probiotics; 2: Buffalo yogurt with *L. acidophilus* LA‐5; 3: Buffalo yogurt with *B. animalis* subsp. *lactis* BB‐12; 4: Cow yogurt without probiotics; 5: Cow yogurt with *L. acidophilus* LA‐5; 6: Cow yogurt with *B. animalis* subsp. *lactis* BB‐12; 7: Sheep yogurt without probiotics; 8: Sheep yogurt with *L. acidophilus* LA‐5; 9: Sheep yogurt with *B. animalis* subsp. *lactis* BB‐12; 10: Goat yogurt without probiotics; 11: Goat yogurt with *L. acidophilus* LA‐5; 12: Goat yogurt with *B. animalis* subsp. *lactis* BB‐12.

Dry matter and fat values of the yogurt samples produced in this study were measured only at the beginning of the storage. The dry matter, fat, and fat‐in‐dry‐matter contents of yogurt samples made from four different types of milk are presented in Figure 2. The highest dry matter and fat values were observed in sheep milk yogurts, while cow milk yogurts exhibited the lowest. Buffalo and sheep milk had higher dry matter and fat contents than cow and goat milk. These results are consistent with previous studies on the subject (Serafeimidou et al. 2013; Yüksel‐Önür 2015; Akbulut‐Çakır and Teker 2021; Hamed et al. 2024).

### 3.2 pH and Titratable Acidity of Yogurts

Throughout the storage period, pH values decreased, and titratable acidity increased in both control and probiotic yogurts made from the four types of milk. The type of milk, storage time, and the use of probiotic cultures had statistically significant effects (*p* < 0.05) on the pH and acidity of the samples (Table 1).

**TABLE 1:** Changes in titratable acidity and pH values of yogurt samples during storage.

	Sample	Storage time (Days)			
		1	7	14	21
Titratable Acidity (%)	1	1.22 ± 0.01^Ddc^	1.34 ± 0.02^Cd^	1.37 ± 0.01^Bc^	1.58 ± 0.01^Ac^
	2	1.22 ± 0.08^Dd^	1.38 ± 0.03^Cc^	1.40 ± 0.01^Bc^	1.62 ± 0.02^Ab^
	3	1.22 ± 0.02^Bdc^	1.26 ± 0.01^Be^	1.27 ± 0.06^Be^	1.49 ± 0.01^Ae^
	4	0.95 ± 0.19^Ci^	0.96 ± 0.01^Ci^	1.12 ± 0.03^Bg^	1.16 ± 0.02^Aj^
	5	0.96 ± 0.08^Dh^	0.99 ± 0.05^Ch^	1.06 ± 0.13^Bh^	1.09 ± 0.13^Ak^
	6	0.99 ± 0.01^Dg^	1.02 ± 0.05^Ch^	1.12 ± 0.10^Bg^	1.14 ± 0.01^Aj^
	7	1.30 ± 0.04^Bb^	1.43 ± 0.09^Cb^	1.50 ± 0.04^Bb^	1.53 ± 0.09^Ad^
	8	1.45 ± 0.08^Da^	1.54 ± 0.13^Ca^	1.60 ± 0.09^Aa^	1.72 ± 0.12^Aa^
	9	1.23 ± 0.02^Bc^	1.26 ± 0.05^Be^	1.31 ± 0.07^Ad^	1.35 ± 0.07^Ag^
	10	1.02 ± 0.01^Df^	1.09 ± 0.01^Cg^	1.19 ± 0.05^Bf^	1.27 ± 0.03^Ah^
	11	1.14 ± 0.03^De^	1.22 ± 0.07^Cf^	1.30 ± 0.01^Bde^	1.39 ± 0.01^Af^
	12	1.00 ± 0.01^Dg^	1.08 ± 0.01^Cg^	1.14 ± 0.10^Bg^	1.20 ± 0.01^Ai^
pH	1	4.28 ± 0.08^Ak^	4.24 ± 0.01^Bi^	4.21 ± 0.013^Ci^	4.11 ± 0.01^Dh^
	2	4.31 ± 0.07^Aj^	4.25 ± 0.05^Bi^	4.25 ± 0.01^Bh^	4.20 ± 0.13^Cf^
	3	4.62 ± 0.01^c^	4.58 ± 0.01^Bc^	4.51 ± 0.09^Cb^	4.47 ± 0.05^Db^
	4	4.27 ± 0.04^Ak^	4.23 ± 0.03^Bj^	4.18 ± 0.06^Cj^	4.14 ± 0.07^Dg^
	5	4.35 ± 0.11^Ai^	4.31 ± 0.12^Bh^	4.28 ± 0.12^Cg^	4.21 ± 0.01^Df^
	6	4.50 ± 0.15^Ae^	4.46 ± 0.09^Be^	4.41 ± 0.05^Cd^	4.32 ± 0.09^Dd^
	7	4.42 ± 0.02^Af^	4.39 ± 0.17^Bf^	4.37 ± 0.01^Ce^	4.34 ± 0.01^Dd^
	8	4.38 ± 0.01^Ag^	4.35 ± 0.14^Bg^	4.34 ± 0.04^Cf^	4.26 ± 0.05^Be^
	9	4.81 ± 0.02^Ab^	4.79 ± 0.03^Bb^	4.77 ± 0.08^Ca^	4.72 ± 0.02^Da^
	10	4.56 ± 0.01^Ad^	4.53 ± 0.10^Bd^	4.47 ± 0.01^Cc^	4.41 ± 0.03^Dc^
	11	4.36 ± 0.01^Ah^	4.32 ± 0.15^Bh^	4.27 ± 0.01^Cg^	4.19 ± 0.01^Df^
	12	4.84 ± 0.07^Aa^	4.82 ± 0.01^Ba^	4.79 ± 0.06^Ca^	4.73 ± 0.05^Da^

*Note:* a–k: Different lower case in the same column and each category indicates a significant difference between the means (*p* < 0.05). A–D: Different upper case in the same row and each category indicates a significant difference between the means (*p* < 0.05). 1: Buffalo yogurt without probiotics; 2: Buffalo yogurt with *L. acidophilus* LA‐5; 3: Buffalo yogurt with *B. animalis* subsp. *lactis* BB‐12; 4: Cow yogurt without probiotics; 5: Cow yogurt with *L. acidophilus* LA‐5; 6: Cow yogurt with *B. animalis* subsp. *lactis* BB‐12; 7: Sheep yogurt without probiotics; 8: Sheep yogurt with *L. acidophilus* LA‐5; 9: Sheep yogurt with *B. animalis* subsp. *lactis* BB‐12; 10: Goat yogurt without probiotics; 11: Goat yogurt with *L. acidophilus* LA‐5; 12: Goat yogurt with *B. animalis* subsp. *lactis* BB‐12.

The pH of all yogurt samples decreased over the storage period. The highest pH values at both the beginning and end of storage were recorded in samples containing *B. animalis* subsp. *lactis* BB‐12 across all milk types. Similar findings were reported by Kurtuldu and Özcan (2018), Soni et al. (2020) and Ayaz et al. (2024). Bifidobacteria are known to possess enzymes capable of metabolizing indigestible oligo‐ and polysaccharides (e.g., xylo‐oligosaccharides, galacto‐oligosaccharides, soybean oligosaccharides, and fructo‐oligosaccharides) (Biavati et al. 2000; Amaretti et al. 2006; de Vrese and Schrezenmeir 2008; Pokusaeva et al. 2011). In addition, it was determined that *B. animalis* subsp. *lactis* BB‐12 showed higher proteolytic activity in milk‐based medium compared to the culture medium. As bifidobacteria ferment lactose and oligosaccharides, their proteolytic activity may increase, and the resulting protein degradation products may buffer the medium to some extent (Abu‐Taraboush et al. 1998; Janer et al. 2005; Bergamini et al. 2009; Ayaz et al. 2024).

The natural acidity of milk is primarily due to components such as casein, phosphate, citrate, albumin, globulin, and dissolved CO_2_. In addition, lactose‐fermenting microorganisms also contribute to increased acidity. Buffalo, sheep, and goat milk naturally have higher acidity than cow milk; hence, yogurts made from these milks also exhibited higher acidity (Yüksel‐Önür 2015). Acidity increased over time due to ongoing fermentation by starter and probiotic bacteria. Similar findings have been reported in studies involving sheep, goat, and cow yogurts Tarchi et al. (2025).

The titratable acidity of all yogurt groups showed a statistically significant increase (*p* < 0.05) throughout the storage period. The lowest acidity values across all storage periods in buffalo, sheep, and goat milk yogurts were recorded in samples inoculated with yogurt culture + *B. animalis* subsp. *lactis* BB‐12. *L. acidophilus* LA‐5 and yogurt starter cultures are homofermentative lactic acid bacteria that primarily produce lactic acid. The acidity values of yogurts containing bifidobacteria are consistent with the results obtained in the studies conducted by Turgut and Cakmakci (2018) and Ayaz et al. (2024).

Although bifidobacteria are not classified as lactic acid bacteria, they are capable of producing lactic acid during carbohydrate fermentation. Unlike conventional lactic acid bacteria, bifidobacteria metabolize lactose via the bifid shunt (fructose‐6‐phosphate phosphoketolase pathway), resulting in the formation of acetic acid and lactic acid at an approximate molar ratio of 3:2 (Biavati et al. 2000; González‐Rodríguez et al. 2013). Since the pKa value of acetic acid (4.76) is approximately one unit higher than that of lactic acid (3.86), acetic acid exhibits nearly tenfold lower acidity at equivalent concentrations (Ballongue 2004; Wang et al. 2014). Consequently, probiotic yogurts containing bifidobacteria tend to display lower overall acidity levels compared to conventional yogurt formulations.

### 3.3 Textural Analysis

To determine the structural properties of the yogurts, water holding capacity, syneresis, viscosity, and texture profile analysis (hardness, adhesiveness, cohesiveness, springiness, and gumminess) were performed.

### 3.4 Water Holding Capacity and Syneresis of Yogurts

Milk type, storage time, and probiotic culture use also significantly affected (*p* < 0.05) the water‐holding capacity (WHC) and syneresis of yogurt samples (Table 2). WHC decreased while syneresis increased over time, in agreement with previous studies on sheep, goat, and cow milk yogurts (Akbulut‐Çakır and Teker 2021). Similar trends were observed for buffalo milk yogurts (Nguyen et al. 2014; Barakat et al. 2021).

**TABLE 2:** Changes in viscosity, hardness, syneresis and water holding capacity of yogurt samples during storage.

Samples		Storage time (Days)								
		1	7	14	21		1	7	14	21
1	Viscosity (cP)	9227.7 ± 63.2^Bb^	9750.0 ± 33.0^Abc^	9883.7 ± 125.8^Ac^	9888.67 ± 82.1^Ac^	Syneresis (%)	0.3 ± 0.02^Dd^	0.5 ± 0.03^Ce^	0.9 ± 0.05^Bd^	1.8 ± 0.08^Ad^
2		9237.7 ± 411.2^Bb^	9735.0 ± 16.9^Abc^	9750.0 ± 50.0^Ac^	9866.67 ± 76.4^Ac^		0.3 ± 0.05^Dd^	0.5 ± 0.02^Ce^	1.0 ± 0.05^Bcd^	1.8 ± 0.04^Ad^
3		8400.3 ± 107.0^Dc^	8681.3 ± 206.9^Cc^	9136.3 ± 77.5^Bd^	9579.3 ± 75.4^Ac^		0.4 ± 0.09^Cd^	0.7 ± 0.05^Bcd^	0.9 ± 0.02^Bd^	1.9 ± 0.01^Acd^
4		5908.3 ± 137.7^De^	6181.67 ± 71.4^Cd^	6591.67 ± 62.7^Be^	6883.3 ± 76.4^Ade^		0.3 ± 0.05^Dd^	0.5 ± 0.11^Ce^	1.0 ± 0.03^Bcd^	2.1 ± 0.04^Acd^
5		6475.3 ± 345.5^Bd^	6525.0 ± 90.1^Bd^	6600.3 ± 93.8^ABe^	7033.3 ± 76.4^Ad^		0.3 ± 0.02^Dd^	0.5 ± 0.01^Ce^	1.0 ± 0.01^Bcd^	2.2 ± 0.12^Acd^
6		5403.0 ± 298.9^Ce^	6050.0 ± 150.0^Bd^	6350.0 ± 73.2^ABe^	6700.0 ± 180.3^Ae^		0.8 ± 0.03^Cc^	0.8 ± 0.04^Cc^	1.2 ± 0.03^Bc^	2.3 ± 0.03^Ac^
7		11725.0 ± 42.0^Ba^	13277.7 ± 57.3^Aa^	14405.7 ± 74.2^Aa^	14488.7 ± 82.1^Aa^		0.3 ± 0.08^Dd^	0.5 ± 0.02^Ce^	0.8 ± 0.11^Bd^	1.8 ± 0.01^Ad^
8		11200.0 ± 47.9^Ca^	12827.7 ± 94.6^Ba^	14355.7 ± 15.6^Aa^	14444.7 ± 75.1^Aa^		0.3 ± 0.01^Dd^	0.5 ± 0.03^Ce^	0.9 ± 0.09^Bd^	1.8 ± 0.03^Ad^
9		9205.7 ± 168.5^Db^	9805.7 ± 58.7^Cb^	10466.7 ± 44.4^Bb^	10789.0 ± 15.0^Ab^		0.3 ± 0.04^Cd^	0.6 ± 0.09^Cde^	0.9 ± 0.09^Bd^	1.9 ± 0.03^Acd^
10		3972.0 ± 554.7^Af^	4255.3 ± 904.2^Ae^	4566.7 ± 428.7^Af^	4977.7 ± 425.0^Af^		1.1 ± 0.01^Cb^	1.4 ± 0.03^Cb^	4.7 ± 0.02^Bb^	9.6 ± 0.01^Ab^
11		3927.7 ± 441.8^Bf^	3922.0 ± 226.3^Be^	4361.0 ± 48.5^ABf^	4683.3 ± 316.5^Af^		1.1 ± 0.06^Cb^	1.5 ± 0.03^Cab^	4.8 ± 0.01^Bb^	9.9 ± 0.01^Ab^
12		2449.7 ± 325.3^Ag^	2661.0 ± 318.1^Af^	2805.6 ± 183.8^Ag^	2822.3 ± 108.1^Ag^		1.5 ± 0.03^Ca^	1.6 ± 0.01^Ca^	5.3 ± 0.02^Ba^	10.8 ± 0.04^Aa^
1	Hardness (g)	258.97 ± 14.54^Bc^	263.23 ± 16.8^ABc^	293.6 ± 6.28^Ac^	289.03 ± 22.0^ABd^	Water holding capacity (%)	75 ± 0.10^Aa^	64 ± 0.02^Bab^	60 ± 0.02^Cab^	56 ± 0.01^Da^
2		239.2 ± 5.27^Ad^	245.63 ± 8.68^Acd^	247.07 ± 14.00^Ad^	255.50 ± 7.90^Ae^		74 ± 0.12^Aa^	66 ± 0.03^Ba^	65 ± 0.04^Ba^	55 ± 0.05^Cab^
3		219.700 ± 0.30^Be^	220.03 ± 2.77^Bd^	242.30 ± 4.33^Ad^	246.03 ± 12.70^Ae^		73 ± 0.01^Aa^	63 ± 0.01^Bb^	63 ± 0.09^Ba^	54 ± 0.02^Cb^
4		172.23 ± 12.73^Af^	171.60 ± 19.43^Ae^	191.77 ± 16.8^ABe^	204.00 ± 3.55^Af^		52 ± 0.06^Ac^	51 ± 0.09^Ade^	50 ± 0.10^Adc^	46 ± 0.11^Be^
5		161.37 ± 12.21^Afg^	162.50 ± 42.69^Ae^	162.80 ± 1.61^Af^	177.40 ± 6.88^Ag^		53 ± 0.07^Ac^	52 ± 0.10^Acd^	49 ± 0.21^Bdc^	46 ± 0.13^Ce^
6		154.00 ± 3.50^Bg^	162.73 ± 5.20^ABe^	161.93 ± 6.88^ABf^	170.57 ± 9.80^Ag^		53 ± 0.13^Ac^	49 ± 0.12^Be^	48 ± 0.31^Bd^	46 ± 0.06^Ce^
7		345.55 ± 2.24^Ba^	360.03 ± 13.26^ABa^	369.93 ± 10.22^Aa^	368.53 ± 9.39^Aa^		56 ± 0.02^Ab^	53 ± 0.01^ABc^	52 ± 0.21^Bbdc^	50 ± 0.04^Cc^
8		330.55 ± 0.72^Cb^	343.86 ± 0.55^Bab^	342.73 ± 0.93^Bb^	351.13 ± 6.99^Ab^		57 ± 0.02^Bb^	53 ± 0.01^Bc^	50 ± 0.07^Cdc^	49 ± 0.12^Ccd^
9		325.30 ± 1.45^Ab^	328.29 ± 1.31^Ab^	329.91 ± 3.00^Ab^	331.74 ± 6.29^Ac^		53 ± 0.01^Ac^	50 ± 0.08^Ae^	59 ± 0.18^Aabc^	47 ± 0.10^Ade^
10		94.41 ± 3.62^Ah^	95.27 ± 0.61^Af^	95.44 ± 0.15^Ag^	95.71 ± 0.86^Ah^		36 ± 0.03^Ad^	33 ± 0.05^Bg^	30 ± 0.04^Ce^	27 ± 0.09 ^Dfg^
11		92.3 ± 0.39^Ah^	91.86 ± 0.27^Af^	92.64 ± 0.69^Ag^	93.26 ± 1.34^Ah^		37 ± 0.10^Ad^	35 ± 0.02^Afg^	30 ± 0.01 ^Be^	27 ± 0.04^Cg^
12		77.17 ± 2.49^Ai^	78.35 ± 2.21^Af^	82.47 ± 7.88^Ag^	81.79 ± 1.05^Ah^		37 ± 0.01^Ad^	36 ± 0.04^Af^	32 ± 0.11^Be^	29 ± 0.13^Cf^

*Note:* a–i: Different lower case in the same column and each category indicates a significant difference between the means (*p* < 0.05). A–D: Different upper case in the same row and each category indicates a significant difference between the means (*p* < 0.05). 1: Buffalo yogurt without probiotics; 2: Buffalo yogurt with *L. acidophilus* LA‐5; 3: Buffalo yogurt with *B. animalis* subsp. *lactis* BB‐12; 4: Cow yogurt without probiotics; 5: Cow yogurt with *L. acidophilus* LA‐5; 6: Cow yogurt with *B. animalis* subsp. *lactis* BB‐12; 7: Sheep yogurt without probiotics; 8: Sheep yogurt with *L. acidophilus* LA‐5; 9: Sheep yogurt with *B. animalis* subsp. *lactis* BB‐12; 10: Goat yogurt without probiotics; 11: Goat yogurt with *L. acidophilus* LA‐5; 12: Goat yogurt with *B. animalis* subsp. *lactis* BB‐12.

Water is primarily retained by the protein matrix in yogurt and the water holding capacity and syneresis are related to the amount of protein (Miocinovic et al. 2016; Nguyen et al. 2018; Li et al. 2022). In our study, the highest WHC and lowest syneresis were observed in buffalo milk yogurts. This can be attributed to the higher total solids and protein content of buffalo milk, particularly its elevated casein and colloidal calcium levels, which promote the formation of a dense and continuous protein network capable of retaining water more effectively (Nguyen et al. 2014; Roy et al. 2020a). In addition, the higher fat content of buffalo milk contributes to improved gel structure and reduced whey separation by filling the protein matrix and enhancing structural stability (Yüksel‐Önür 2015; Barakat et al. 2021). Under acidic conditions, the strong network structure formed by the interaction of denatured whey proteins with casein micelles hinders syneresis and consequently increases the water holding capacity (Tables 1 and 2).

Interestingly, sheep milk yogurts, despite having higher dry matter content, exhibited lower WHC at the beginning of storage than buffalo milk yogurts. This can be explained by the higher fat content and lower non‐fat dry matter (and thus protein content) of sheep milk yogurts (Figure 2). Increased acidity also contributes to reduced WHC and increased syneresis (Table 2) (Park et al. 2007; Nguyen et al. 2018; Tarchi et al. 2025).

Goat milk yogurts had the lowest WHC and highest syneresis throughout storage. Goat milk contains less casein and more serum proteins and non‐protein nitrogen than cow, sheep, and buffalo milk, resulting in a weaker yogurt structure (Park et al. 2007; Miocinovic et al. 2016). Other studies conducted with cow, sheep, buffalo, camel and goat milk yogurts have similarly found goat milk yogurts to have the lowest WHC and highest syneresis (Joon et al. 2017; Khalifa and Zakaria 2019; Vianna et al. 2019).

### 3.5 Viscosity and TPA Analysis

To determine the textural properties of yogurts, in addition to WHC and syneresis, viscosity and Texture Profile Analysis (TPA) parameters were also evaluated.

Viscosity and hardness of all yogurt samples increased over time. These parameters were significantly affected (*p* < 0.05) by milk type, storage time, and probiotic use (Table 2), which aligns with findings from previous research (Salvador and Fiszman 2004; Domagala 2009; Saccaro et al. 2009; Akgun et al. 2017; Vianna et al. 2019; Tarchi et al. 2025).

Viscosity increases due to interactions between denatured serum proteins and casein micelles during heat treatment. Starter‐induced acidification further enhances these interactions (Anema et al. 2004). Under acidic conditions, heat‐denatured serum proteins, particularly β‐lactoglobulin, interact more effectively with κ‐casein on the micelle surface through hydrophobic interactions and disulfide bond formation. This results in the development of a denser and more continuous protein network, thereby increasing resistance to flow and leading to higher viscosity values, as observed in Table 1 (Anema et al. 2004; Vianna et al. 2019; Tarchi et al. 2025).

Sheep milk has higher casein and colloidal calcium content than other milks, while buffalo milk proteins have greater thermal resistance. Hence, sheep yogurts showed the highest viscosity and hardness, followed by buffalo, cow, and goat yogurts. Goat milk, with lower casein content and larger micelle size, produced yogurts with the lowest viscosity and hardness (Park et al. 2007; Yüksel‐Önür 2015; Vianna et al. 2019; Roy et al. 2020b). These findings agree with prior studies showing sheep yogurts having the highest hardness (Domagala 2009), and buffalo yogurts having higher viscosity than cow yogurts (Nguyen et al. 2014).

Cohesiveness and springiness values generally declined over storage time. Within each milk type, yogurts with different cultures had similar values. Goat milk yogurts exhibited the highest cohesiveness and springiness, followed by sheep, cow, and buffalo yogurts (Table 3). Similar results were reported in a study conducted with sheep, cow, and goat milk yogurts (Tarchi et al. 2025).

**TABLE 3:** Changes in cohesiveness, gumminess and springiness values of yogurt samples during storage.

		Sample	Storage time (Days)			
			1	7	14	21
Cohesiveness	Buffalo	1	0.57 ± 0.02^Abc^	0.50 ± 0.02^Bbc^	0.48 ± 0.02^Bde^	0.46 ± 0.04^Bd^
		2	0.59 ± 0.04^Aab^	0.50 ± 0.02^Bbc^	0.52 ± 0.03^Bc^	0.48 ± 0.01^Bd^
		3	0.54 ± 0.02^Acd^	0.52 ± 0.02^ABb^	0.50 ± 0.01^BCcde^	0.48 ± 0.02^Cd^
	Cow	4	0.49 ± 0.01^Ae^	0.48 ± 0.02^Ac^	0.48 ± 0.02^Ae^	0.48 ± 0.02^Ad^
		5	0.51 ± 0.02^Ade^	0.49 ± 0.02^Abc^	0.51 ± 0.01^Acd^	0.49 ± 0.02^Ad^
		6	0.50 ± 0.00^Be^	0.52 ± 0.00^Abc^	0.51 ± 0.01^ABc^	0.51 ± 0.01^Ac^
	Sheep	7	0.60 ± 0.01^Aab^	0.59 ± 0.03^Aa^	0.57 ± 0.01^Ab^	0.57 ± 0.01^Ab^
		8	0.60 ± 0.01^Aab^	0.59 ± 0.02^Aa^	0.58 ± 0.00^Aab^	0.59 ± 0.00^Aab^
		9	0.59 ± 0.02^Aab^	0.59 ± 0.00^Aa^	0.60 ± 0.00^Aab^	0.60 ± 0.02^Aa^
	Goat	10	0.60 ± 0.02^Aab^	0.59 ± 0.01^Aa^	0.59 ± 0.01^Aab^	0.60 ± 0.00^Aa^
		11	0.61 ± 0.02^Aa^	0.60 ± 0.02^Aa^	0.60 ± 0.01^Aab^	0.59 ± 0.00^Aa^
		12	0.60 ± 0.01^Aa^	0.60 ± 0.02^Aa^	0.61 ± 0.00^Aa^	0.61 ± 0.01^Aa^
Gumminess (g)	Buffalo	1	146.74 ± 10.8^Ac^	132.67 ± 5.26^Ac^	141.22 ± 9.54^Ac^	133.63 ± 2.24^Ad^
		2	140.38 ± 7.77^Ac^	122.6 ± 2.46^Bcd^	128.24 ± 6.77^Bd^	123.67 ± 3.67^Be^
		3	119.27 ± 4.94^Ad^	114.66 ± 5.05^Ad^	120.89 ± 2.90^Ad^	117.81 ± 2.04^Af^
	Cow	4	84.90 ± 5.92^Be^	83.06 ± 9.65^Be^	91.05 ± 7.30^ABe^	98.81 ± 1.29^Ag^
		5	82.52 ± 3.56^Ae^	80.43 ± 23.96^Ae^	82.33 ± 0.94^Af^	86.30 ± 3.92^Ah^
		6	77.27 ± 2.16^Be^	83.99 ± 3.03^ABe^	82.55 ± 4.31 ^ABf^	87.73 ± 4.04^Ah^
	Sheep	7	206.30 ± 4.03^Aa^	212.88 ± 9.44^Aa^	212.04 ± 4.88^Aa^	212.05 ± 4.97^Aa^
		8	198.77 ± 3.13^ABa^	201.7 ± 6.65^ABa^	200.03 ± 2.42^Ab^	206.27 ± 4.61^Ab^
		9	192.08 ± 7.29^Ab^	192.90 ± 3.32^Ab^	197.68 ± 2.70^Ab^	200.26 ± 3.68^Ac^
	Goat	10	56.36 ± 0.88^Af^	56.51 ± 1.32 ^Af^	56.40 ± 0.93^Ag^	57.82 ± 0.25^Aı^
		11	56.10 ± 1.58^Af^	55.49 ± 2.02 ^Af^	55.54 ± 0.76^Ag^	56.27 ± 085^Aı^
		12	46.62 ± 1.97^Ag^	46.97 ± 2.76 ^Af^	49.89 ± 4.56^Ag^	50.00 ± 1.31^Aj^
Springiness	Buffalo	1	146.74 ± 10.8^Ac^	132.67 ± 5.26^Ac^	141.22 ± 9.54^Ac^	133.63 ± 2.24^Ad^
		2	140.38 ± 7.77^Ac^	122.6 ± 2.46^Bcd^	128.2 ± 6.77^Bd^	123.7 ± 3.67^Be^
		3	119.27 ± 4.94^Ad^	114.66 ± 5.05^Ad^	120.89 ± 2.90^Ad^	117.81 ± 2.04^Af^
	Cow	4	84.90 ± 5.92^Be^	83.06 ± 9.65^Be^	91.05 ± 7.30^ABe^	98.81 ± 1.29 ^Ag^
		5	82.52 ± 3.56^Ae^	80.43 ± 23.96^Ae^	82.33 ± 0.94^Af^	86.30 ± 3.92^Ah^
		6	77.27 ± 2.16^Be^	83.99 ± 3.03^ABe^	82.55 ± 4.31^ABf^	87.73 ± 4.04 ^Ah^
	Sheep	7	206.30 ± 4.03^Aa^	212.88 ± 9.44^Aa^	212.04 ± 4.88^Aa^	212.05 ± 4.97^Aa^
		8	198.77 ± 3.13^ABa^	201.70 ± 6.6^ABa^	200.03 ± 2.42^Ab^	206.27 ± 4.61^Ab^
		9	192.08 ± 7.29^Ab^	192.90 ± 3.32^Ab^	197.68 ± 2.70^Ab^	200.26 ± 3.68^Ac^
	Goat	10	56.36 ± 0.88^Af^	56.51 ± 1.32^Af^	56.40 ± 0.93^Ag^	57.82 ± 0.25^Aı^
		11	56.10 ± 1.58^Af^	55.49 ± 2.02^Af^	55.54 ± 0.76^Ag^	56.27 ± 085^Aı^
		12	46.62 ± 1.97^Ag^	46.97 ± 2.76^Af^	49.89 ± 4.56^Ag^	50.00 ± 1.31^Aj^

*Note:* a–h: Different lower case in the same column and each category indicates a significant difference between the means (*p* < 0.05). A–D: Different upper case in the same row and each category indicates a significant difference between the means (*p* < 0.05). 1: Buffalo yogurt without probiotics; 2: Buffalo yogurt with *L. acidophilus* LA‐5; 3: Buffalo yogurt with *B. animalis* subsp. *lactis* BB‐12; 4: Cow yogurt without probiotics; 5: Cow yogurt with *L. acidophilus* LA‐5; 6: Cow yogurt with *B. animalis* subsp. *lactis* BB‐12; 7: Sheep yogurt without probiotics; 8: Sheep yogurt with *L. acidophilus* LA‐5; 9: Sheep yogurt with *B. animalis* subsp. *lactis* BB‐12; 10: Goat yogurt without probiotics; 11: Goat yogurt with *L. acidophilus* LA‐5; 12: Goat yogurt with *B. animalis* subsp. *lactis* BB‐12.

Cohesion reflects the internal binding strength of a food matrix, while elasticity indicates elasticity after the deterioration force is removed (Di Monaco et al. 2008; Mousavi et al. 2019). Goat milk, which is rich in serum proteins and medium‐chain fatty acids and has smaller casein micelles, produces more cohesive and elastic yogurts due to increased protein‐water and protein–protein interactions (Maathuis et al. 2017; Monteiro et al. 2019; Daszkiewicz et al. 2023).

Gumminess, which is related to hardness and viscosity, was highest in sheep milk yogurts, followed by buffalo, cow, and goat milk yogurts (Table 3) (Park et al. 2007; Peleg 2019). These findings are consistent with previous studies showing buffalo yogurts as gummier than cow yogurts (Jooyandeh et al. 2022), and cow yogurts as gummier than goat yogurts (Joon et al. 2017; Terzioğlu and Bakirci 2024). Probiotic cultures significantly reduced gumminess in yogurts made from all milk types. This trend was also observed by Bonczar et al. (2002) for sheep milk yogurts and Terzioğlu and Bakirci (2024) for cow and goat milk yogurts.

### 3.6 Microbiological Analysis

**FIGURE 3:**
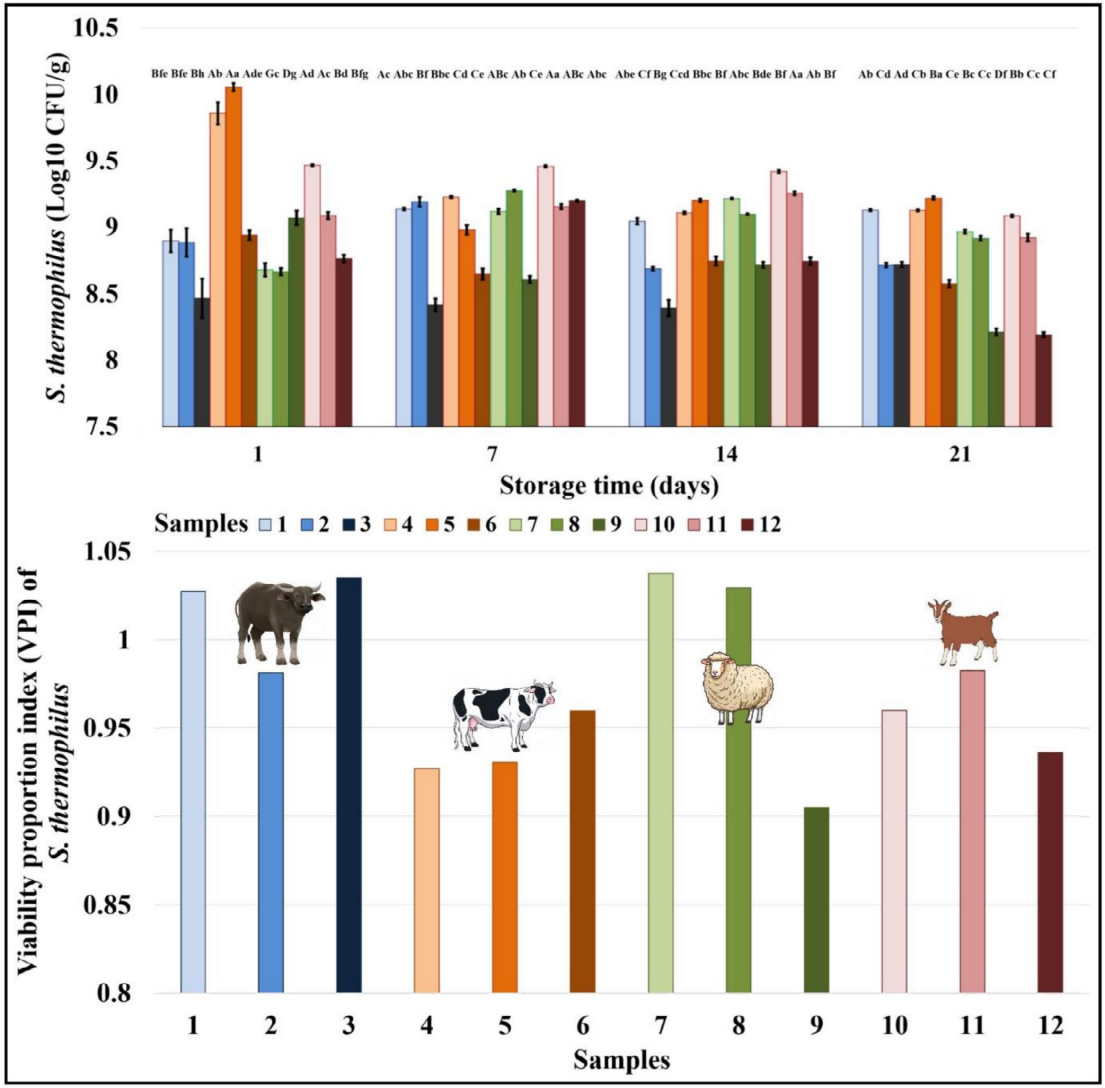
*S. thermophilus* counts and viability proportion index (VPI) of yogurt samples (a–h) Different lower case indicates a significant difference between different samples at the same storage time (*p* < 0.05). (A–G) Different upper case a significant difference between the same samples at different storage times (*p* < 0.05). 1: Buffalo yogurt without probiotics; 2: Buffalo yogurt with *L. acidophilus* LA‐5; 3: Buffalo yogurt with *B. animalis* subsp. *lactis* BB‐12; 4: Cow yogurt without probiotics; 5: Cow yogurt with *L. acidophilus* LA‐5; 6: Cow yogurt with *B. animalis* subsp. *lactis* BB‐12; 7: Sheep yogurt without probiotics; 8: Sheep yogurt with *L. acidophilus* LA‐5; 9: Sheep yogurt with *B. animalis* subsp. *lactis* BB‐12; 10: Goat yogurt without probiotics; 11: Goat yogurt with *L. acidophilus* LA‐5; 12: Goat yogurt with *B. animalis* subsp. *lactis* BB‐12.

Microbial analysis of yogurt samples was conducted throughout storage. *S. thermophilus* counts fluctuated but generally decreased over time (Figure 3). Similar results were reported for fermented milks produced from cow and goat milk and stored for 28 days (Dimitrellou et al. 2019), yogurts produced from cow, sheep and goat milk and stored for 28 days (Vianna et al. 2019) and set‐type yogurts (Birollo et al. 2000). Nevertheless, all samples‐maintained counts above 8 log CFU/g at the end of storage, satisfying the Codex Alimentarius (2022) minimum threshold for fermented dairy products (Codex STAN 243‐2003).

In the relevant standard, yogurt is defined as a dairy product that is formed as a result of the fermentation of milk sugar by symbiotic yogurt bacteria and contains at least 10^7^ CFU/g live starter at the end of fermentation (Codex Alimentarius 2022). This high lactic acid bacteria viability is associated with various health benefits, including improved lactose digestion (Arsov et al. 2022; Zhou et al. 2024), intestinal microbiota regulation (Chen et al. 2024; El‐Salam et al. 2025), treatment of nonalcoholic fatty liver disease (Nabavi et al. 2014; Zhang et al. 2024), antimicrobial activity (Munteanu‐Ichim et al. 2024; Wiktorczyk‐Kapischke et al. 2024), immune system support (Hasegawa and Bolling 2023; Zou et al. 2023; Gholamhosseinpour et al. 2024), antioxidant activity (Pyo et al. 2005; Gholamhosseinpour et al. 2024), anti‐tumor effect (Ciric et al. 2023; Nami et al. 2024), and anti‐cholesterolemic effect (Abd El‐Gawad et al. 2005; Liu et al. 2006).

**FIGURE 4:**
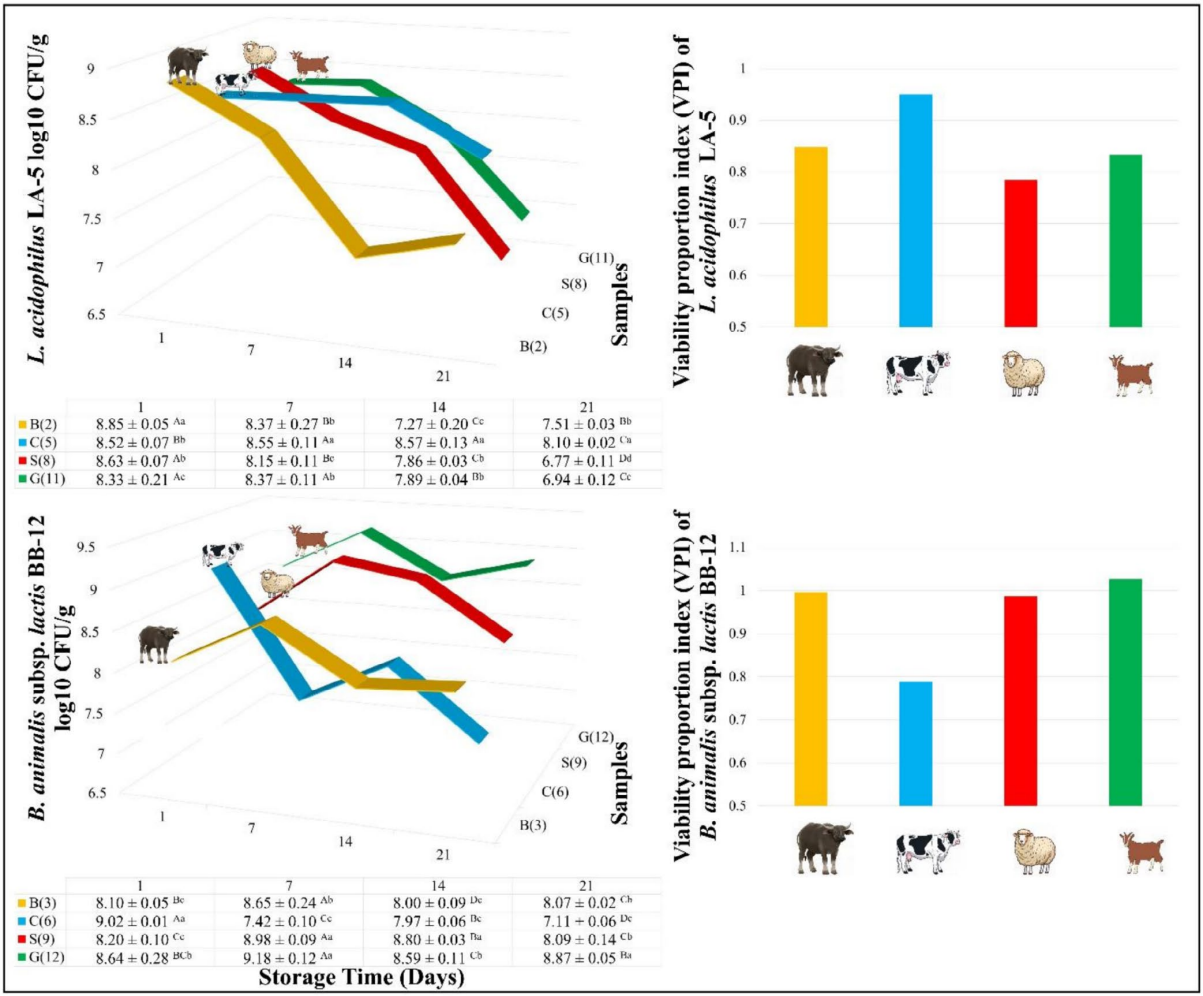
Probiotic counts and viability proportion index (VPI) of yogurt samples (a–d): Different lower case indicates a significant difference between different samples at the same storage time (*p* < 0.05). (A–D) Different upper case indicates a significant difference between the same samples at different storage times (*p* < 0.05). 1: Buffalo yogurt without probiotics; 2: Buffalo yogurt with *L. acidophilus* LA‐5; 3: Buffalo yogurt with *B. animalis* subsp. *lactis* BB‐12; 4: Cow yogurt without probiotics; 5: Cow yogurt with *L. acidophilus* LA‐5; 6: Cow yogurt with *B. animalis* subsp. *lactis* BB‐12; 7: Sheep yogurt without probiotics; 8: Sheep yogurt with *L. acidophilus* LA‐5; 9: Sheep yogurt with *B. animalis* subsp. *lactis* BB‐12; 10: Goat yogurt without probiotics; 11: Goat yogurt with *L. acidophilus* LA‐5; 12: Goat yogurt with *B. animalis* subsp. *lactis* BB‐12.

*L. acidophilus* LA‐5 counts decreased in all samples over time (Figure 4). At the end of storage, cow milk yogurts had the highest LA‐5 counts, while sheep milk yogurts had the lowest. Counts remained above 7 log CFU/g in buffalo and cow milk yogurts for the full 21 days, but dropped below this threshold in sheep and goat milk yogurts. The values obtained for LA‐5 in the study are supported by the results obtained in similar yogurt studies. In some of these studies, it was determined that LA‐5 numbers decreased over time in cow and buffalo milk yogurts and fell below the 7‐log value, while they increased in sheep milk yogurts (Beheshtipour et al. 2012; Sarvari et al. 2014; Vianna et al. 2019; Barakat et al. 2021).

*B. animalis* subsp. *lactis* BB‐12 counts decreased in buffalo, cow, and sheep yogurts over time, but increased in goat milk yogurts. Throughout storage, BB‐12 counts in all samples remained above 7 log CFU/g (Figure 4). The growth of BB‐12 in goat milk can be attributed to its high lactose and oligosaccharide content (Park et al. 2007; de Vrese and Schrezenmeir 2008; Bode 2012; Oliveira et al. 2015), and favorable physicochemical properties such as appropriate pH, titratable acidity, and acidification capacity (Galina et al. 2007), making goat milk an effective probiotic carrier. It has been reported in various studies that *B. animalis* subsp. lactis BB‐12 counts in yogurts produced from different milks and fermented milks decrease over time (Beheshtipour et al. 2012; Sarvari et al. 2014; Varga et al. 2014; Ranadheera et al. 2016; Kurtuldu and Özcan 2018; Prasanna and Charalampopoulos 2019; Lucatto et al. 2020; Szajnar et al. 2020; Barakat et al. 2021). In a comparative study conducted with cow, goat, and soy milk, similar to the results in our study, the highest growth rate for *B. animalis* subsp. *lactis* BB‐12 was found in goat milk and the lowest growth rate was found in soy milk (Slačanac et al. 2013). For a product to be classified as probiotic, a viable count of at least 10^6^–10^7^ CFU/g is required (Bedani et al. 2013). All yogurt samples in this study met this criterion and can therefore be considered probiotic yogurts.

### 3.7 Sensory Analysis

**FIGURE 5:**
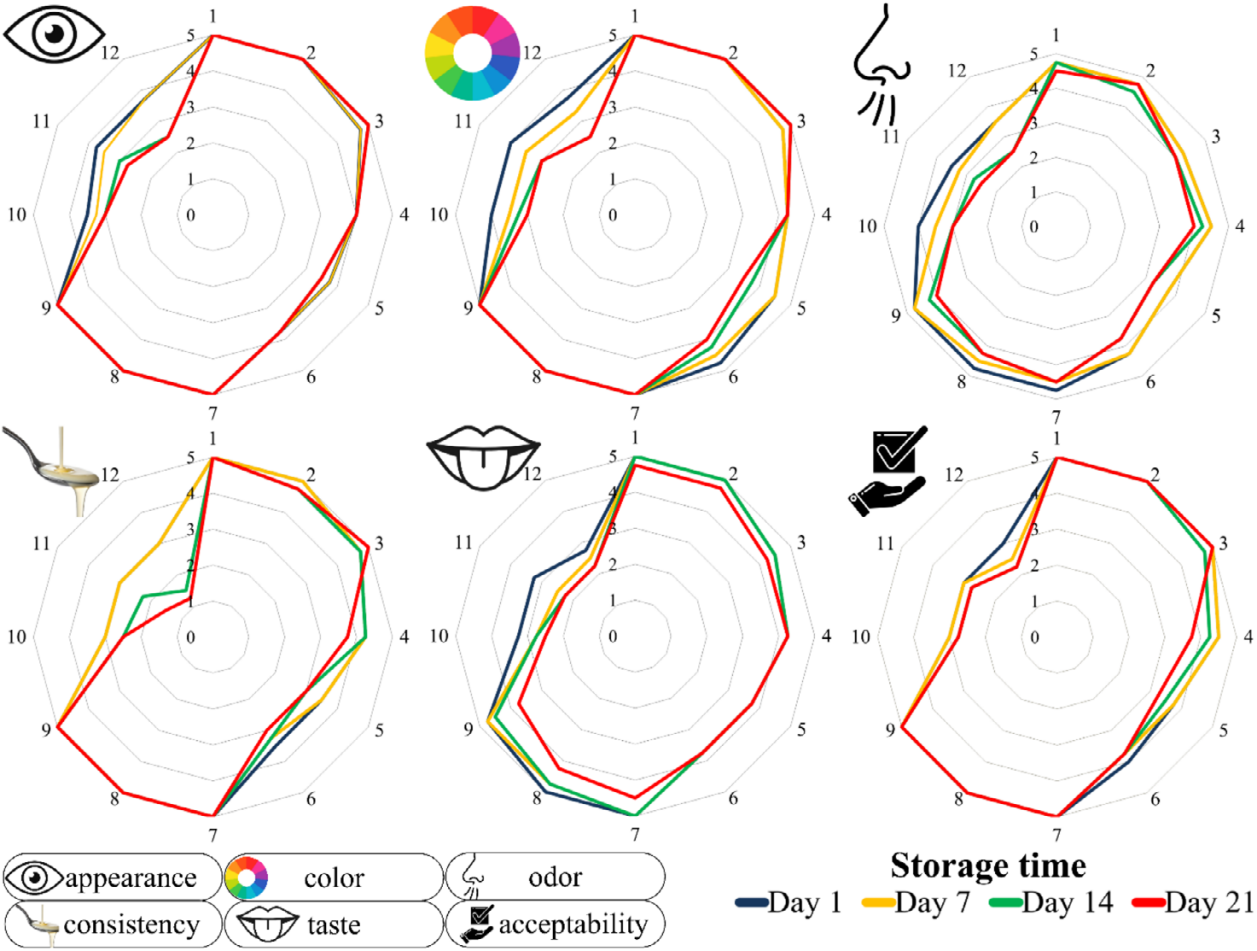
Sensory scores of yogurt samples with and without probiotics. 1: Buffalo yogurt without probiotics; 2: Buffalo yogurt with *L. acidophilus* LA‐5; 3: Buffalo yogurt with *B. animalis* subsp. *lactis* BB‐12; 4: Cow yogurt without probiotics; 5: Cow yogurt with *L. acidophilus* LA‐5; 6: Cow yogurt with *B. animalis* subsp. *lactis* BB‐12; 7: Sheep yogurt without probiotics; 8: Sheep yogurt with *L. acidophilus* LA‐5; 9: Sheep yogurt with *B. animalis* subsp. *lactis* BB‐12; 10: Goat yogurt without probiotics; 11: Goat yogurt with *L. acidophilus* LA‐5; 12: Goat yogurt with *B. animalis* subsp. *lactis* BB‐12.

In the sensory evaluation, appearance, color, odor, consistency, taste, and overall acceptability were assessed. Sheep and buffalo milk yogurts received the highest scores, while goat milk yogurts scored the lowest (Figure 5). The weak structure of goat milk due to its protein ratios (Jenness 1980; Miocinovic et al. 2016) and the presence of medium‐chain fatty acids such as caproic, caprylic, and capric acid, which negatively affect taste and aroma (Jenness 1980; Agnihotri and Prasad 1993), caused its scores to be low.

Previous studies have reported that mixing goat milk with sheep milk improves sensory quality of yogurts (Matos et al. 2013). Ahmed et al. (2014) found that buffalo yogurts had higher sensory scores than cow and goat yogurts. In our study, control group yogurts generally received higher scores than probiotic yogurts. The acetic acid produced by bifidobacteria via the bifid shunt likely contributed to the lower flavor scores in probiotic yogurts (Biavati et al. 2000; González‐Rodríguez et al. 2013). This aligns with previous findings indicating that bifidobacteria can negatively impact the taste, aroma, and texture of yogurts without affecting their color or appearance or aroma (Hekmat and Reid 2006; Soni et al. 2020).

### 3.8 Correlation Analysis Between Physicochemical Properties, Textural Attributes, and Starter and Probiotic Viability

**FIGURE 6:**
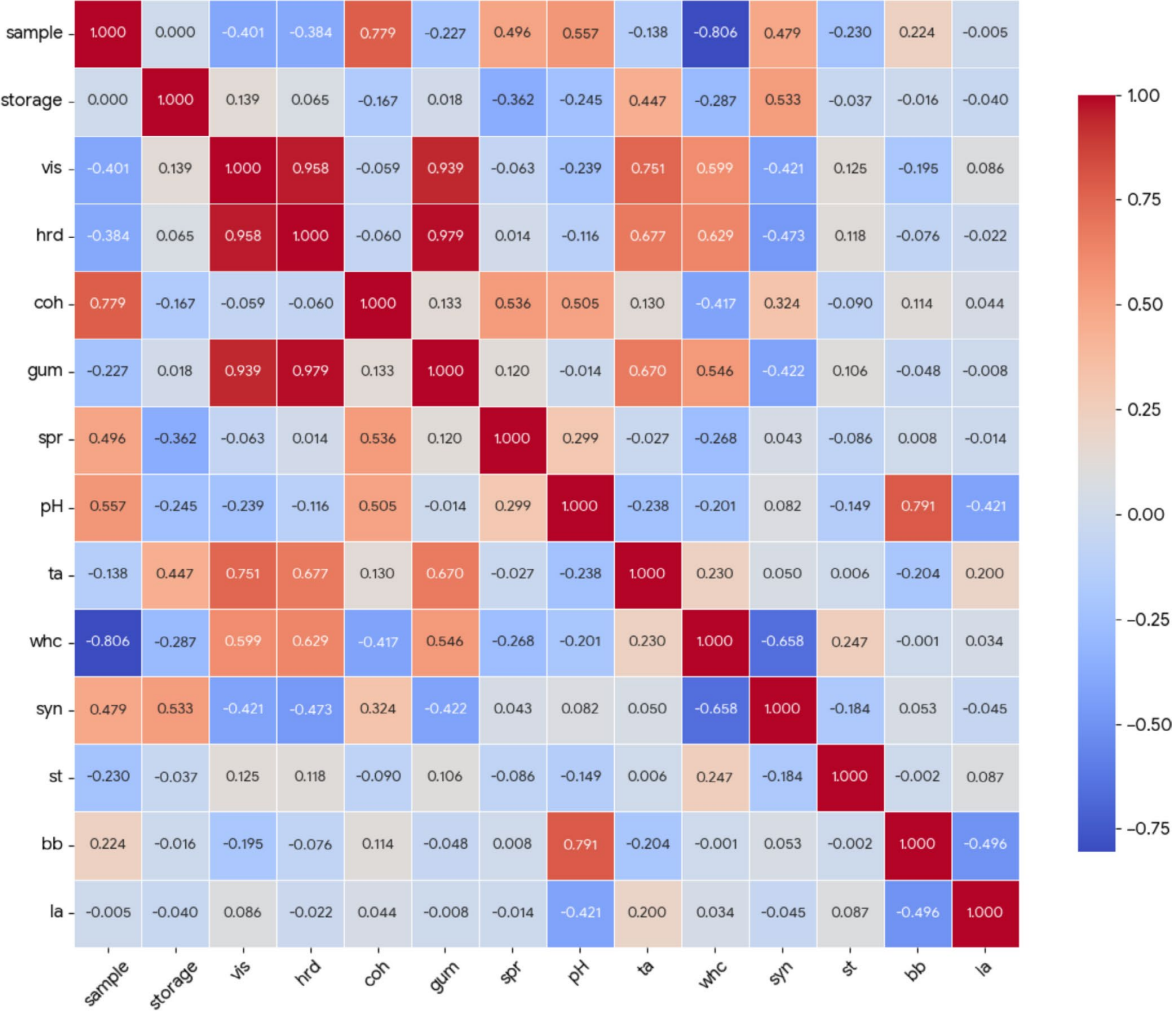
Heatmap visualization of the correlation matrix of chemical, textural, and microbial characteristics of the yogurt samples.bb, *B. animalis* subsp. *lactis* BB‐12; coh, cohesiveness; gum, gumminess; hrd, hardness; la, *L. acidophilus* LA‐5; spr, springiness; st, *S. thermophilus* ; syn, syneresis; ta, titratable acidity; vis, iscosity; whc, water holding capacity.

The Pearson correlation heatmap (Figure 6) illustrates the interrelationships among physicochemical parameters, textural attributes, and microbial characteristics of the yogurt samples. Strong positive correlations were observed between viscosity and hardness (*r* > 0.95, *p* < 0.01), indicating that the development of a firm gel structure was closely associated with increased resistance to flow. Hardness was also strongly and positively correlated with gumminess (*r* > 0.85, *p* < 0.01), which is consistent with the definition of gumminess as a composite parameter largely dependent on gel firmness and internal structural strength. These relationships confirm that textural parameters evolved in a coordinated manner as the protein network became more compact.

Water‐holding capacity (WHC) exhibited a strong negative correlation with syneresis (*r* < −0.65, *p* < 0.01), demonstrating that improved water retention within the protein matrix effectively limited whey separation. In addition, titratable acidity showed negative associations with springiness and gumminess, suggesting that progressive acidification weakened the elastic and cohesive characteristics of the yogurt gel. This finding supports the role of acid‐induced protein aggregation in promoting firmness while simultaneously reducing elasticity.

Regarding microbial parameters, *S. thermophilus* counts showed moderate associations with textural and physicochemical variables, indicating that starter activity contributed indirectly to gel formation through acidification. In contrast, probiotic viability ( *L. acidophilus* LA‐5 and *B. animalis* subsp. *lactis* BB‐12) exhibited weak or negligible correlations with most textural parameters, suggesting that probiotic survival was more strongly influenced by the compositional and microstructural characteristics of the yogurt matrix rather than by individual texture attributes. Overall, the correlation patterns observed in Figure 6 highlight the central role of physicochemical interactions—particularly protein‐water and protein–protein interactions—in governing yogurt texture, while indicating a limited direct coupling between probiotic viability and mechanical properties.

## 4 Conclusion

The present study demonstrates that the type of milk and probiotic strain exert strong interactive effects on the compositional, rheological, and microbiological properties of yogurt. Among the milks examined, sheep and buffalo milk produced yogurts with superior textural and sensory attributes, while goat milk provided the most suitable environment for maintaining *B. animalis* subsp. *lactis* BB‐12 viability during storage. These findings suggest that the compositional matrix of milk, particularly its protein, fat, and oligosaccharide content, governs both the technological behavior and probiotic functionality of yogurt. From a technological standpoint, blending milk types with complementary compositional features (e.g., sheep + goat or buffalo + cow) may optimize the structural integrity and consumer acceptability of probiotic yogurts.

Future research should incorporate metabolomic and volatile compound analyses to elucidate molecular mechanisms underlying probiotic–milk interactions and to design next‐generation functional dairy products with enhanced health‐promoting potential.

## Author Contributions

**Seval Andiç:** conceptualization, investigation, methodology, resources, supervision, writing – review and editing. **Şehriban Oğuz:** investigation, methodology, writing – review and editing. **Şevval Şevgin Demirhan:** investigation.

## Funding

We would like to thank the Scientific Research Projects Coordination Unit of Van Yuzuncu Yil University (Turkey) for the financial support provided for this project (FYL‐2022‐9979).

## Conflicts of Interest

The authors declare no conflicts of interest.

## Data Availability

The data that support the findings of this study are available from the corresponding author upon reasonable request.
